# Clinical insomnia among elderly primary care attenders in Wuhan, China: A multicenter cross-sectional epidemiological study

**DOI:** 10.3389/fpubh.2022.1026034

**Published:** 2022-10-21

**Authors:** Bao-Liang Zhong, Hong-Jie Li, Yan-Min Xu, Xue-Feng Jiang

**Affiliations:** ^1^Department of Psychiatry, Wuhan Mental Health Center, Wuhan, China; ^2^Department of Clinical Psychology, Wuhan Hospital for Psychotherapy, Wuhan, China; ^3^Department of Outpatient and Emergency, Shanghai Mental Health Center, Shanghai Jiao Tong University School of Medicine, Shanghai, China

**Keywords:** insomnia, elderly, primary care, epidemiology, China

## Abstract

**Background and objectives:**

Integrating sleep health into primary care is a promising approach to narrow the treatment gap for insomnia in older adults but data regarding the epidemiological characteristics of insomnia among elderly primary care attenders (EPCAs) are very limited. This study examined the prevalence and correlates of clinical insomnia among Chinese EPCAs.

**Methods:**

By using two-stage consecutive sampling method, a total of 757 EPCAs were recruited from seven urban and six rural primary care centers in Wuhan, China. The Insomnia Severity Index (ISI) and the Geriatric Depression Scale (15 item version) were administered to assess insomnia severity and depressive symptoms, respectively.

**Results:**

The two-week prevalence of clinical insomnia (ISI score ≥ 15) was 28.9%. Significant correlates of clinical insomnia were: female sex (vs. male, OR = 2.13, *P* < 0.001), fair and poor family relationship (vs. good, OR = 1.59, *P* = 0.028), hypertension (OR = 1.67, *P* = 0.004), heart disease (OR = 1.73, *P* = 0.048), arthritis (OR = 2.72, *P* = 0.001), and depressive symptoms (OR = 4.53, *P* < 0.001).

**Conclusion:**

The high prevalence of clinical insomnia among Chinese EPCAs suggests a high level of sleep health need in older patients in China's primary care settings. Considering the many negative outcomes associated with insomnia, it is necessary to integrate sleep health into primary care in China.

## Introduction

While insomnia is prevalent among the general population, in particular among the elderly population, it is often under-recognized, under-diagnosed, and under-treated (1–4). For example, in a recently published population-based survey in Hebei, China, as high as 9.4 and 21.9% of the residents aged 18–59 years and 60 years and older suffered from insomnia symptoms, respectively, as defined by a cut-off score of seven or greater on the Athens Insomnia Scale (AIS) (5). However, the corresponding rates of visiting a doctor for sleep problems in the past year among individuals with insomnia of the two age-groups were as low as 5.5 and 6.8%, respectively (6). In an international survey across four high-income countries (France, Italy, Japan, and the USA), overall, 15.8% of the adults with a history of insomnia had consulted a physician about their sleep problems, 10.6% of those who consulted physicians were prescribed a drug for the sleep problems, and 6.2% of those who were prescribed drugs were still taking these drugs at the time of this survey (7). Taken together, these data suggest the high level of unmet needs in insomnia management in both the adult and elderly population in China and other countries in the world.

Given the wide availability and easy accessibility of primary care services in most countries in the world (8, 9), primary care physicians (PCPs) are in a unique position to detect older adults with sleep problems and initiate treatment at an early stage when it may be more cost-effective. In the case of China, because of its limited mental health service resources and their unequal distribution between urban and rural regions, and older adults' preference for seeking health services at primary care settings, particularly rural older adults, integrating sleep health into primary care has been a very promising approach to narrow the treatment gap for insomnia and other common mental health problems in older adults (10–13). To facilitate the planning and provision of sleep healthcare services in primary care settings, it is necessary to have the knowledge on the epidemiological characteristics of insomnia among elderly primary care attenders (EPCAs) in China.

In comparison to the numerous studies on insomnia in community-dwelling older adults (4, 14, 15), studies examining insomnia in EPCAs have been very limited. To date, three studies have investigated the prevalence and correlates of insomnia in older adults attending primary care, of which only one was conducted in China (16–18). By using DSM-III-R, Hohagen and colleagues assessed the presence of insomnia in a sample of 330 older patients from the offices of five general practitioners in Mannheim, Germany, and found a 23% prevalence of insomnia and its three significant correlates in older general practice patients: female sex, depression, and organic brain syndrome (16). In Kerala, India, Dahale and colleagues used the Insomnia Severity Index (ISI) to examine insomnia in 1,574 EPCAs from 71 primary care centers and found that 11.8% of the EPCAs suffered from clinical insomnia (17). Factors associated with insomnia in EPCAs in this study included older age, female sex, chronic medical illness, common mental disorders, and greater disability (17). In an urban primary care center in Shanghai, China, Xu and colleagues used the Pittsburgh Sleep Quality Index (PSQI) to screen for insomnia in a volunteer sample of 90 EPCAs and found the insomnia prevalence was 53.9%; however, this study did not analyze correlates of insomnia (18). Limitations of the only available study in China include the convenient sampling, the small sample size, and no inclusion of rural EPCAs. Further, although PSQI is often used to screen for insomnia with reasonable accuracy, it has been criticized for lack of specificity and its problematic factor construct and, strictly speaking, it is only suitable for the discrimination between “good” and “poor” sleepers, not individuals with and without insomnia (19–23). Therefore, the epidemiology of insomnia in Chinese EPCAs remains largely unknown.

To fill the above knowledge gaps, the current study was set out to investigate the prevalence and correlates of clinical insomnia in EPCAs from both urban and rural primary care centers in Wuhan, the largest city with more than 10 million residents in central China (24, 25).

## Methods

### Participants and sampling

Between October 2015 and November 2016, we conducted a large-scale cross-sectional survey among 791 EPCAs in seven urban and six rural primary care centers in Wuhan, China (8, 10, 26, 27). The outcomes of interest of this study included quality of life, common mental health problems, psychosocial problems, and insomnia. The present study focused on insomnia. Participants were recruited *via* two-stage cluster consecutive sampling. In brief, the first stage purposively selected 13 primary care centers from the 13 districts (seven urban and six rural) in Wuhan, one center each district, which were located in or nearest to the most populous area of the district. The second stage consecutively invited older patients who were 65 years old or over and seeking treatment at these primary care centers during the survey period of this study to participate. Patients who refused to participate and were not able to complete the survey due to severe physical illnesses and cognitive impairment and psychotic symptoms were excluded.

Before the fieldwork of this survey, the study protocol was approved by the Institutional Review Board of Wuhan Mental Health Center. All participants provided written informed consent before the interview.

### Questionnaire and procedures

The survey instrument was a questionnaire, which was specifically developed for this study and was administered by trained PCPs of the selected primary care centers in a face-to-face interview format.

Socio-demographic variables in the questionnaire were age, sex, education, marital status, self-rated financial status (good, fair, poor), residence place, living arrangement (alone or with others), and self-rated relationship with family members (good, fair, poor).

Lifestyle factors included smoking and physical exercise. Current smokers were those who were currently smoking at least one cigarette per day on at least 5 days per week (10). Respondents participating in physical exercise regularly were those having the habit of physical exercise (27).

A checklist was used to ascertain the presence of six chronic medical conditions: hypertension, diabetes mellitus, heart disease, cerebrovascular disease, chronic obstructive pulmonary disease (COPD), and arthritis.

The validated Chinese Geriatric Depression Scale, 15-item version (GDS-15), was used to assess depressive symptoms (28–30). The total score of the GDS-15 is the sum of the 15 items, with a possible range of 0–15 and a cut-off score of five or more indicating clinically significant depressive symptoms in China (31).

We used the validated Chinese ISI to evaluate insomnia in the past 2 weeks, which is developed according to the DSM-IV diagnostic criteria for insomnia and consists of seven items scoring from “0 = none” to “4 = very severe” (32, 33). The ISI total score ranges from zero to 28, with ≥ 15 being considered as clinical insomnia (23, 34).

### Statistical analysis

Prevalence of clinical insomnia was calculated. Chi-square test was used to compare rates of insomnia between/across subgroups according to characteristics such as sex and education levels. Binary logistic regression analysis with the forward selection (Wald) method was used to examine the independent correlates of insomnia. Statistically significant variables from the Chi-square test were included into the multiple logistic regression model. Odds Ratios (ORs) and their 95% confidence intervals (CIs) were used to quantify the associations between insomnia and correlates. The statistical significance level was set at *P* < 0.05 (two-sided). SPSS software version 15.0 package (SPSS Inc., Chicago, IL, USA) was used for all analyses.

## Results

In total, 757 EPCAs completed the survey questionnaire. The mean age of the study participants was 72.78 years (standard deviation = 5.99, range = 65–97) and 407 (53.8%) were women. Characteristics of the study sample and prevalence rates of clinical insomnia by variables are shown in Table 1.

**Table 1:** Characteristics of the sample of elderly primary care attenders and prevalence rates of clinical insomnia by sample characteristics.

**Characteristics**		**No. of older adults**	**No. of older adults with insomnia symptoms**	**Prevalence (%)**	**χ^2^**	***P***
Sex	Male	350	74	21.1	19.199	<0.001
	Female	407	145	35.6		
Age (years)	65–74	490	146	29.8	0.507	0.477
	75+	267	73	27.3		
Education	Illiterate	177	67	37.9	14.457	0.001
	Primary school	216	69	31.9		
	Middle school and above	364	83	22.8		
Marital status	Married	529	149	28.2	0.498	0.480
	Others*	228	70	30.7		
Self-rated financial status	Good	133	30	22.6	8.808	0.012
	Fair	536	153	28.5		
	Poor	88	36	40.9		
Residence place	Urban	409	112	27.4	1.034	0.309
	Rural	348	107	30.7		
Living alone	No	673	195	29.0	0.006	0.939
	Yes	84	24	28.6		
Self-rated family relationship	Good	596	158	26.5	7.981	0.005
	Fair and poor**	161	61	37.9		
Currently smoking	No	637	190	29.8	1.574	0.210
	Yes	120	29	24.2		
Habit of physical excise	No	319	90	28.2	0.138	0.710
	Yes	438	129	29.5		
Hypertension	No	390	92	23.6	11.158	0.001
	Yes	367	127	34.6		
Diabetes	No	642	179	27.9	2.259	0.133
	Yes	115	40	34.8		
Heart disease	No	678	186	27.4	7.075	0.008
	Yes	79	33	41.8		
Stroke and other cerebrovascular diseases	No	693	199	28.7	0.183	0.669
	Yes	64	20	31.3		
Chronic obstructive pulmonary disease	No	713	200	28.1	4.615	0.032
	Yes	44	19	43.2		
Arthritis	No	700	190	27.1	14.441	<0.001
	Yes	57	29	50.9		
Depressive symptoms	No	522	98	18.8	84.355	<0.001
	Yes	235	121	51.5		

*“Others” included never married, separated, divorced, widowed, cohabitating, and remarried.

**Because of the very small numbers of the category of “poor” relationship (n <10), “poor” and “fair” were merged into one category.

In total, 219 elderly patients (28.9%) had clinical insomnia (ISI score ≥ 15) during the past 2 weeks.

Results from Chi-square test (Table 1) display that significantly higher rates of clinical insomnia were observed in women (vs. men), in illiterate patients (vs. middle school and above), in patients with poor financial status (vs. good), in patients having poor and fair family relationship (vs. good), in patients suffering from hypertension, in patients suffering from heart disease, in patients suffering from COPD, in patients suffering from arthritis, and in patients having depressive symptoms (*P* ≤ 0.032).

Six significant correlates of clinical insomnia were identified (Table 2): female sex (vs. male, OR = 2.13, *P* < 0.001), fair and poor family relationship (vs. good, OR = 1.59, *P* = 0.028), hypertension (OR = 1.67, *P* = 0.004), heart disease (OR = 1.73, *P* = 0.048), arthritis (OR = 2.72, *P* = 0.001), and depressive symptoms (OR = 4.53, *P* < 0.001).

**Table 2:** Correlates of clinical insomnia in elderly primary care attenders.

**Factor**	**Risk level**	**Reference level**	**Coefficient**	**Standard error**	**χ^2^**	***P***	**OR (95%CI)**
Sex	Female	Male	0.757	0.183	17.154	<0.001	2.13 (1.49,3.05)
Self-rated family relationship	Fair and poor*	Good	0.462	0.211	4.809	0.028	1.59 (1.05,2.40)
Hypertension	Yes	No	0.512	0.180	8.073	0.004	1.67 (1.17,2.38)
Heart disease	Yes	No	0.548	0.277	3.924	0.048	1.73 (1.01,2.98)
Arthritis	Yes	No	1.001	0.310	10.440	0.001	2.72 (1.48,4.99)
Depressive symptoms	Yes	No	1.512	0.182	69.056	<0.001	4.53 (3.17,6.48)

*Because of the very small numbers of the category of “poor” relationship (n <10), “poor” and “fair” were merged into one category.

## Discussion

To the best of our knowledge, this is the first study in China that examined the epidemiological characteristics of insomnia in older adults attending primary care centers. The main findings are the 28.9% prevalence of clinical insomnia in EPCAs and six correlates of insomnia in this patient population: female sex, fair and poor family relationship, hypertension, heart disease, arthritis, and depressive symptoms.

In population-based studies using ISI and the same definition of clinical insomnia, the prevalence rates of clinical insomnia in rural community-residing older adults and older adults during the COVID-19 pandemic in China were 19.2 and 15.6%, respectively (22, 35). Compared to these prevalence estimates in the elderly population, we found a much higher prevalence of clinical insomnia in Chinese EPCAs. The 28.9% prevalence of insomnia in Chinese EPCAs is also much higher than the 11.8% prevalence of insomnia in India EPCAs (17). Because both older age and major medical conditions are major risk factors for insomnia in population-based studies (5, 35, 36), the elevated risk of insomnia in Chinese EPCAs might be primarily due to the older age and poor physical health of this older and physically ill population. In addition, we speculated that the very low treatment rate of insomnia in the elderly population in China might partly explain the high risk of insomnia in EPCAs (6).

In line with previous studies (15–17, 35, 37), we found the greater risk of insomnia in EPCAs who were women, had fair and poor family relationship, and were depressed. The higher prevalence of insomnia in females may be ascribed to their more vulnerability to negative socioeconomic factors and stressful life events in comparison to males (38). Further, women are more likely to develop common mental health problems, including depression and anxiety, which could result in elevated risk of insomnia in women (37). The close relationship between depression and insomnia has been consistently reported in various populations including the EPCAs (16, 17, 35, 39). The reciprocal relationship between depression and insomnia might be explained by their shared pathophysiological pathways, including gut microbiome composition, genetic overlap, and neurotransmitter system in the brain (40). The traditional Chinese culture values filial piety and family harmony, which are significant determinants of mental wellbeing in older Chinese adults (41, 42). Because Confucianism is deeply rooted in values, beliefs, and attitudes of older Chinese adults (43), the significant association of insomnia with fair and poor family relationship, an indicator of family disharmony, is expected.

The etiology of insomnia is multifactorial and involves various social, mental, and physical factors (15). In the literature, medical conditions linked with insomnia include cancer, heart disease, high blood pressure, diabetes, arthritis, chronic pain, and gastrointestinal problems (44–46). Our findings on the significant associations of insomnia with hypertension, heart disease, and arthritis are consistent with prior studies. The physical discomfort, pain, and feelings of depression and stress caused by major medical conditions may explain the elevated risk of insomnia in EPCAs with hypertension, heart disease, and arthritis.

This study has a few limitations. First, this is a cross-sectional study, so the causality between identified correlates and insomnia cannot be inferred in EPCAs. Longitudinal data are warranted to answer this clinical question. Second, we did not assess the recognition and treatment of insomnia in EPCAs by PCPs and older patients' attitudes toward sleep health services provided in general practice, which are important for the planning of sleep health services in Chinese primary care centers. Third, some factors associated with insomnia such as social support, chronic pain, and environmental noise were not measured in this study.

In conclusion, nearly 30% of the Chinese EPCAs suffer from clinical insomnia, suggesting a high level of sleep health need in older patients in China's primary care settings. Considering the many negative outcomes associated with insomnia and the inadequate mental health service resources in China (25), it is necessary to integrate sleep health into primary care. Sleep health services for Chinese EPCAs need to include periodic screening for insomnia, expanded psychosocial supports, effective management of major medical conditions and depression, and, when necessary, referral to psychiatrists and sleep specialists. Further, services for EPCAs would be more effective if they targeted those who are women, have fair and poor family relationship, suffer from several chronic medical conditions such as hypertension and arthritis, and are depressed.

## Data availability statement

The original contributions presented in the study are included in the article/supplementary material, further inquiries can be directed to the corresponding author.

## Ethics statement

The studies involving human participants were reviewed and approved by the Institutional Review Board of Wuhan Mental Health Center (approval number WMHC-IRB-S065). The patients/participants provided their written informed consent to participate in this study.

## Author contributions

B-LZ: acquisition and analysis of data for the study, drafting the article, and interpretation of data for the study. H-JL and Y-MX: design and acquisition of data for the study. X-FJ and B-LZ: drafting the article, revising the article for important intellectual content, and interpretation of data for the study. All authors contributed to the article and approved the submitted version.

## Funding

This work was supported by National Natural Science Foundation of China (Grant Number: 71774060), 2015 Irma and Paul Milstein Program for Senior Health Awards from the Milstein Medical Asian American Partnership Foundation, the Young Top Talent Programme in Public Health from Health Commission of Hubei Province (PI: B-LZ), and Wuhan Health and Family Planning Commission (Grant Number: WX18C12, WX17Q30, WG16A02, and WG14C24). The funding source listed had no role in study design; in the collection, analysis, and interpretation of data; in the writing of the report; and in the decision to submit the paper for publication.

## Conflict of interest

The authors declare that the research was conducted in the absence of any commercial or financial relationships that could be construed as a potential conflict of interest.

## Publisher's note

All claims expressed in this article are solely those of the authors and do not necessarily represent those of their affiliated organizations, or those of the publisher, the editors and the reviewers. Any product that may be evaluated in this article, or claim that may be made by its manufacturer, is not guaranteed or endorsed by the publisher.
